# A Hybrid Approach of Using Symmetry Technique for Brain Tumor Segmentation

**DOI:** 10.1155/2014/712783

**Published:** 2014-03-09

**Authors:** Mubbashar Saddique, Jawad Haider Kazmi, Kalim Qureshi

**Affiliations:** ^1^Department of Computer Science, COMSATS Institute of Information Technology, Abbottabad, Pakistan; ^2^Department of Information Science, College of Computing Sciences and Engineering, Kuwait University, Kuwait

## Abstract

Tumor and related abnormalities are a major cause of disability and death worldwide. Magnetic resonance imaging (MRI) is a superior modality due to its noninvasiveness and high quality images of both the soft tissues and bones. In this paper we present two hybrid segmentation techniques and their results are compared with well-recognized techniques in this area. The first technique is based on symmetry and we call it a hybrid algorithm using symmetry and active contour (HASA). In HASA, we take refection image, calculate the difference image, and then apply the active contour on the difference image to segment the tumor. To avoid unimportant segmented regions, we improve the results by proposing an enhancement in the form of the second technique, EHASA. In EHASA, we also take reflection of the original image, calculate the difference image, and then change this image into a binary image. This binary image is mapped onto the original image followed by the application of active contouring to segment the tumor region.

## 1. Introduction

Digital image processing has found its applications in medical image analysis and researchers are finding more and more ways to help the physician and surgeons in the complex process of image analysis needed for diagnosis. Special importance is the area of medical image segmentation and analysis. During diagnosis, usually, a specific part of body is imaged using one of the many medical imaging modalities (MRI, X-rays, CT scan, etc.). These images are then analyzed by the human observers (physicians and surgeons) to obtain clues about the problem. Tumor and related abnormalities constitute a major cause of disability and death worldwide. Detection and classification of tumor are not only complex but also expensive. To get detailed information about the anatomy of human soft tissues, an advanced medical imaging technique, called magnetic resonance imaging (MRI), is used. MRI gives different information about those structures in the body which are otherwise observable with an X-ray, ultrasound, or computed tomography (CT) scan, but the advantage of MRI is the higher quality of its images and lack of side effects on the body tissues.

MRI employs a magnetic field and pulses of radio wave energy to make pictures of organs and structures inside the body. The problem is, however, the amount of the resultant data which is too much to be analyzed manually. This constitutes a main hurdle in the effective use of MRI and obligates the use of computer-aided automatic or semiautomatic techniques to analyze the product images. In this regard, image segmentation is always considered to be effective enough to play a vital role in MRI based diagnosis. The goal of segmentation is getting the information from the image that is more meaningful and easier to analyze.

Many segmentation techniques are there, in the literature, for brain MRI images but they suffer from many problems. These techniques can segment the tumor but alongside they may segment some other unimportant regions too. Secondly they are limited to find the tumor in one side of the brain, either left or right. Thirdly, before applying the algorithm, the position of tumor should be known, that is, if it is on the right side or left. Fourthly, they are not that good in finding multiple tumors, if they exist. Lastly, most of them require user interaction.

In this paper we present two hybrid segmentation techniques and later on compare our result with an existing technique [1]. Our techniques address the problems described above. The first technique is based on symmetry and we call it a hybrid algorithm using symmetry and active contour (HASA). In HASA, we take reflection image, calculate the difference image, and then apply active contour on the difference image to segment the tumor. To avoid unimportant segmented regions, we improve the results by proposing an enhancement in the form of the second technique, EHASA. In EHASA, we also take reflection of the original image, calculate the difference image, and then change this image into a binary image. This binary image is mapped onto the original image followed by the application of active contouring to segment the tumor region.

The rest of the paper is organized as follows. In Section 2, we briefly describe the related work and highlight some advantages and disadvantages of the different techniques.  Section 3 presents the proposed scheme, while Section 4 outlines some experimental results.  Section 5 concludes the paper.

## 2. Related Work

Liu [5] categorized segmentation as boundary based, region based, and hybrid [6]. The boundary-based techniques rely on the concept of snake or active contour [7, 8]. Region-based technique may be data driven or knowledge driven. The data driven techniques can further be classified as supervised and unsupervised.

Supervised methods are based on the manual labeling of the training data. Techniques, like neural networks or support vector machine (SVM), are used in supervised segmentation. Many researchers have worked on supervised segmentation; for example, one such method employs a SVM [2] which is currently used for binary classification. According to Schmidt (http://webdocs.cs.ualberta.ca/~btap/Papers/dana2007.pdf), supervised methods have the advantage to perform different task simply by changing the training set. In addition, the tumor is detected automatically when the learning is complete. But supervised methods suffer from the overheads of special training and the time/delay involved in its acquisition.

On the other hand, unsupervised segmentation methods, e.g. thresholding and region growing, do not require any special training. Many works can be found in the literature dealing with these techniques, such as [3], which utilizes first threshold intensities on some manually selected area and then use a region growing algorithm to expand the thresholded region to the edges defined by the Sobel edge detection filter. Despite having no need of training, the unsupervised methods are handicapped by the manual selection for region growing and prespecification of the number of regions. Besides, they are mainly restricted to such simple tasks where there exists some obvious indicator of abnormality, for example, the presence of a contrast agent. Lastly, the tumors have no clearly defined intensities with these methods.

Knowledge driven techniques are also called registration based segmentation techniques. In these techniques, prior knowledge about the anatomical structure of the tissues is needed for segmentation. Gering [4] takes a database of the normal brain as a reference to match and diagnose a brain tumor but the problem he faced was the possibility of intensity non-standardization which may make comparisons very difficult. To overcome this problem, the author proposed to utilize the characteristic of symmetry because brain is symmetrical and its left and right halves can be easily compared to find the abnormality. Kause [9] presented a hybrid type of method which employs template registration and statistical classification (Kth nearest neighbor—KNN) to segment a homogeneous type of brain tumor. The pros and cons of supervised, unsupervised, and registration based segmentation are summarized in Table 1.

Symmetry is an important characteristic to identify the structure of the object and has been used in many fields. Of special interest is the line or bilateral symmetry which has got attention of many researchers. Works exist in the literature to find the symmetrical axis on an image [10–13]. The work of Atallah [10] requires the objects to be presented as lines, circles, and points. The author applied some morphological operations like thinning or grass fire but only on binary images. Jiao [14] have presented a simple method to find a symmetry line. The author finds the edge map first and from this edge map calculates edge centroid, *G*_*i*_, by using the following formula:

(1) $$\begin{array}{c} G_{i}=\frac{1}{k}\sum_{j=1}^{k}P_{i,j},\;P_{i,j}\in P_{e}, \end{array}$$

where *G*_*i*_, is the abscissa of *i*th line. The least square method is then employed to get the symmetry line. In [14], the authors give a method to locate the tumor but with most tumors the boundaries are not well defined and many nontumor regions are also segmented alongside. Ray et al. [15] have also proposed a symmetry based method, with the idea of a bounding box, but the technique only locates tumor when it is on either side of the brain, whether left or right. In other words, if both sides have tumors, then the method does not locate it. Same is the case with multiple tumors. The symmetry based localization method of Mancas [16] simply find the histogram of the parts on left and right of a median line M and obtain a curve S from the difference of the two histogram. If the curve is deviated at some point/window from the horizontal line of symmetry, then asymmetry is present which points to some abnormality, otherwise there is symmetry meaning normal brain tissue. Khotanlou [17] also segment the brain tumor by employing fuzzy logic and symmetry and then utilize the deformable model to enhance the segmentation. The features comparison of supervised, unsupervised, and registration-based symmetry techniques are shown in Table 2.

## 3. The Proposed Technique

As has been discussed above, supervised techniques require prior knowledge and user interaction for segmentation. Similarly the unsupervised and registration based techniques also require prior knowledge. We propose a hybrid solution which does not require prior knowledge and also segment the tumor better than the above mentioned techniques. Symmetry is one of the most important characteristics of vision. It is a fast and high level approach to object understanding. An object has a line or bilateral symmetry if the two halves, resulting from the partition of the object along the line, are replica of each other.

An object having exactly one line of symmetry can be termed as zygomorphic. Our brain can be classified as a zygomorphic object, since it is symmetric along a line, drawn vertically on the image. With reference to the human body, if bilateral symmetry is violated, then it may be due to some abnormalities, most of the times. Hence, normal brain is symmetric but if some abnormality is present, then it may become asymmetric.

### 3.1. A Hybrid Algorithm Using Symmetry and Active Con-Tour (HASA)

The HASA pseudocodes are presented in Figure 2(c). Following are the main steps involved.

**Figure 1:**
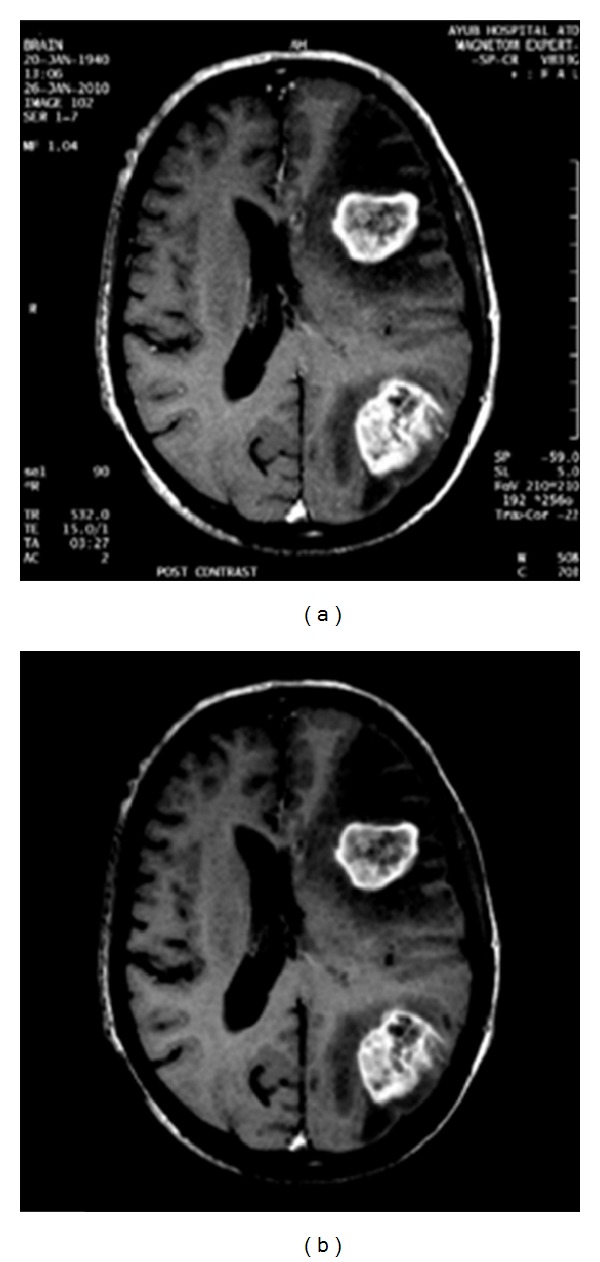
Original and morphological image output; (a) original image *O*(*x*, *y*); (b) image after morphological operation.

**Figure 2:**
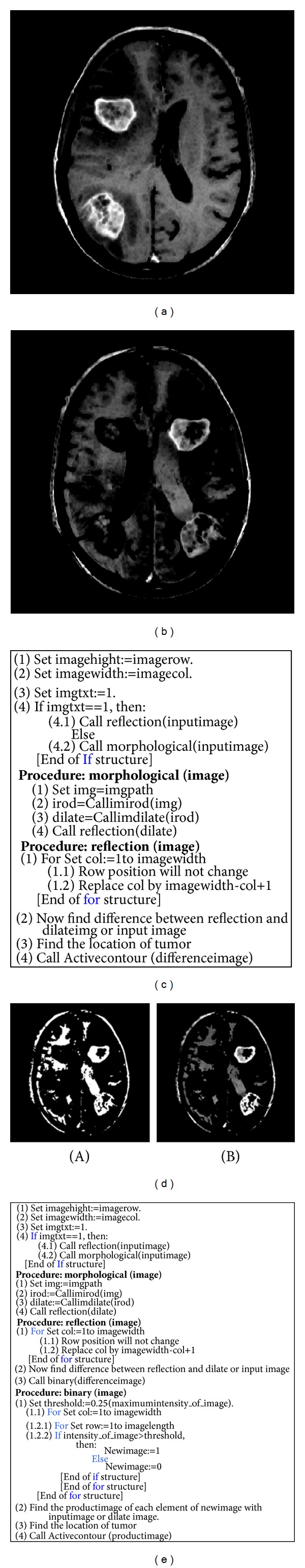
(a) Reflection Image *R*(*x*, *y*) of *O*(*x*, *y*). (b) New image *D*(*x*, *y*). (c) Pseudocode of HASA technique. (d) (A) Mask of *D*(*x*, *y*) and (B) product of mask and *O*(*x*, *y*). (e) Pseudocode of EHASA technique.

(1) Read the image; if image required preprocessing, then first apply morphological operations erosion and dilation. We get the resultant image as shown in Figures 1(a) and 1(b).
(2) Find the reflection of the original image *O*(*x*, *y*).
  (a) Find the size of image that is row and col.
  (b) Find the reflection image, *R*(*x*, *y*), of the image *O*(*x*, *y*).
(3) Find the difference image, *D*(*x*; *y*), which is obtained by the following equation:
  (2) $$\begin{array}{c} D\left(x,y\right)=O\left(x,y\right)-R\left(x,y\right), \end{array}$$
  where *D*(*x*, *y*) is new image, *O*(*x*, *y*), and *R*(*x*, *y*) are original and reflection image, respectively. New image is shown in Figure 2(b).
(4) Find the location where maximum numbers of higher intensities are aggregated.
(5) Apply active contouring [5] to the location, found in step (4), to get the final result in the form of segmented tumor.

### 3.2. Enhance Hybrid Algorithm Using Symmetry and Active Contour (EHASA)

In EHASA enhanced technique, we use threshold value for making binary image and then map this binary image on original image so that we can get lesser parts when we apply active contouring. EHASA pseudocodes are shown in Figure 2(e). The steps are as follows.

(1) Apply morphological operations, like erosion and dilation, if preprocessing needed. After this optional step let our image is denoted by *O*(*x*, *y*).
(2) Find the reflection image, *R*(*x*; *y*), of the image *O*(*x*; *y*).
(3) Find the difference image, *D*(*x*; *y*), which is obtained by the following equation:
  (3) $$\begin{array}{c} D\left(x,y\right)=O\left(x,y\right)-R\left(x,y\right). \end{array}$$
(4) Threshold *D*(*x*, *y*) to get a binary image. The value of the threshold *T* is about 25% of the maximum intensity in *O*(*x*, *y*), that is,
  (4) $$\begin{array}{c} T=\mathrm{Max}\left(O\left(x;y\right)\right)\vee 0.25. \end{array}$$
(5) Now find the mask of *D*(*x*, *y*) using the threshold value
   If (*D*(*x*, *y*) > avg)
     
*D*(*x*, *y*) = 1.
   Else
     
*D*(*x*, *y*) = 0.
(6) Map the mask with original image *O*(*x*, *y*); that is, multiply the mask and original image *O*(*x*, *y*).
(7) Apply active contouring [1] to the location, found in step (4), to get the final result in the form of segmented tumor.

## 4. Results and Discussion

**Figure 3:**
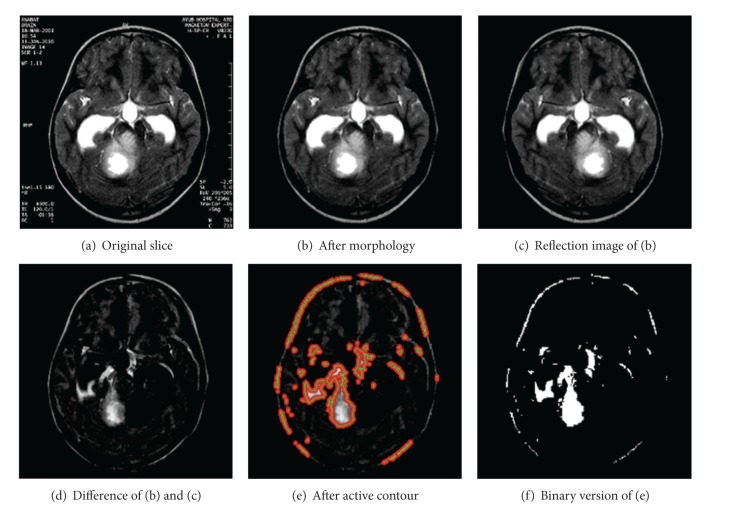
Application of HASA.

We have applied the proposed method to DICOM format MRI data of 20 different patients. The results obtained with one such example, when subjected to HASA, are shown in Figure 3. Part (a) of the figure is the original DICOM image which, after morphological preprocessing, yields the image in Figure 3(b). The refection image of the image got after preprocessing is illustrated in Figure 3(c).  Figure 3(d), which shows the difference image between Figures 3(b) and 3(c), is subjected to active contouring to find the boundary of the tumor and the results are evident in Figure 3(e).  Figure 3(f) is simply the binary image of Figure 3(e). The segmented tumor is shown in Figure 3(f).

**Figure 4:**
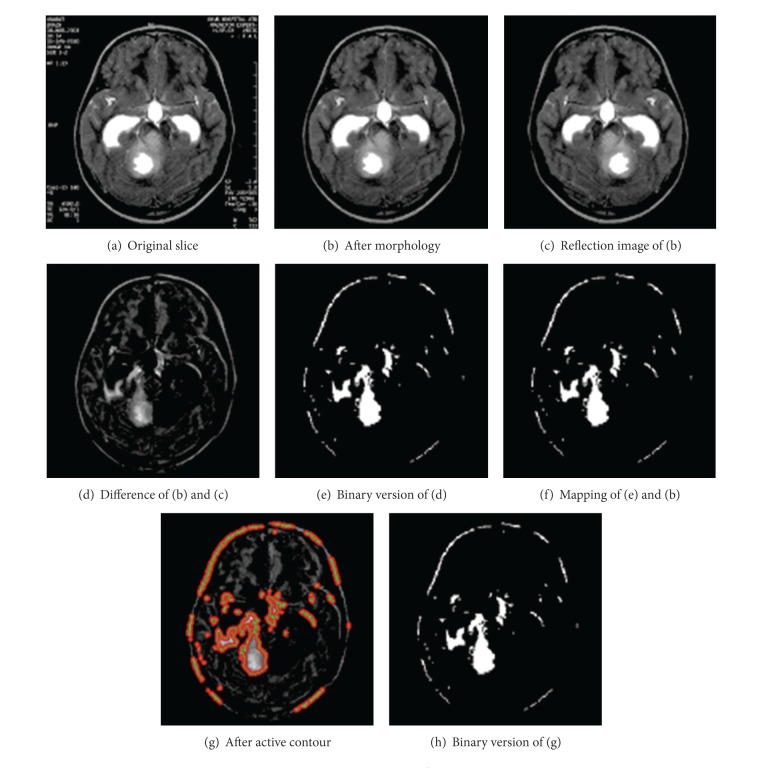
Application of EHASA.

During the segmentation process, in HASA, we got some extra segmented regions. To overcome these, we applied our 2nd technique (EHASA) on the same image data which resulted in the images shown in Figure 4.  Figure 4(a) is the original image and after applying the morphological operations, that is, erosion and dilation, we got the image in Figure 4(b). We next found the refection of image Figure 4(b), which is shown in Figure 4(c).  Figure 4(d) shows the difference image between Figure 4(b) and Figure 4(c). After applying a threshold *T*, on the image of Figure 4(d), we got the binary image in Figure 4(e).  Figure 4(f) depicts the mapping of binary image Figure 4(e) on the image in Figure 4(b). After applying the active contour on the image, given in Figure 4(f), we got the result shown in Figure 4(g).  Figure 4(h) is a binary representation of Figure 4(g).

**Figure 5:**
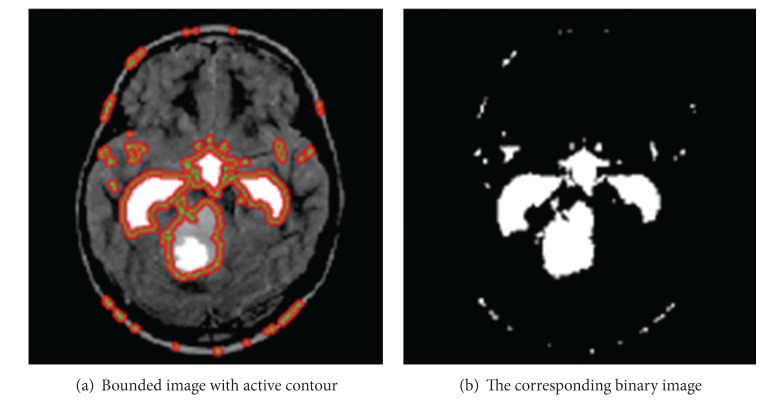
Application of Chan-Vese method.

**Figure 6:**
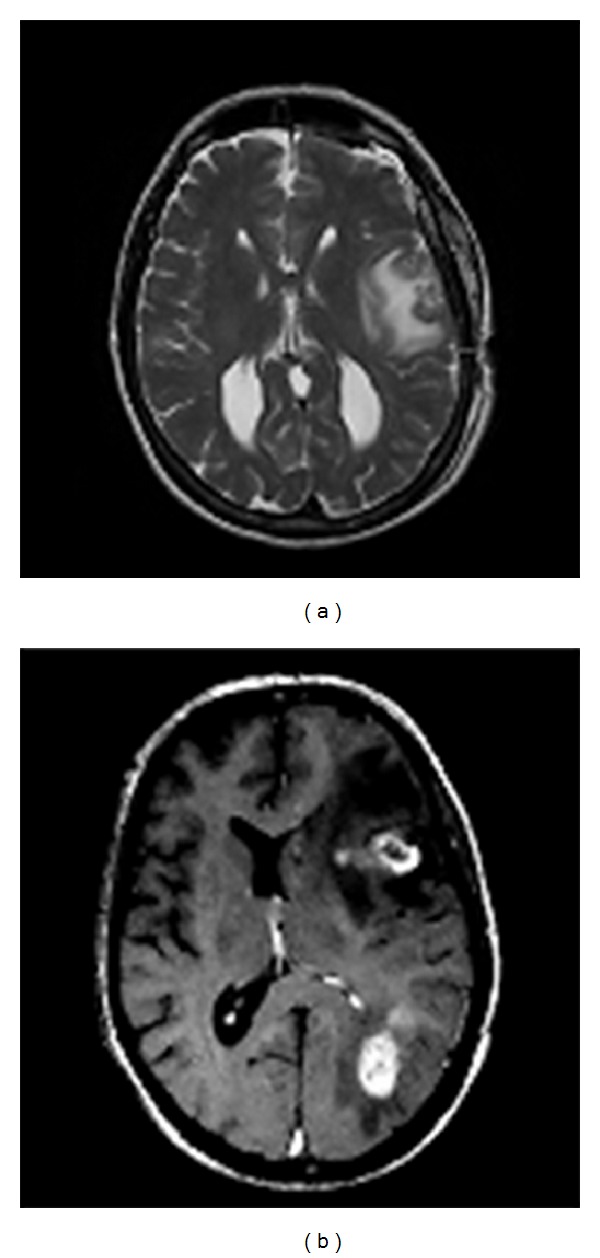
The two original images.

**Figure 7:**
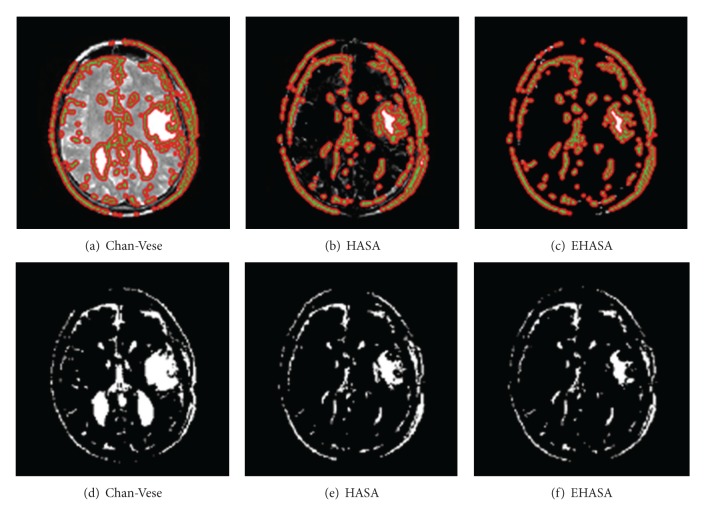
Result after the application of different techniques on input images shown in Figure 6(a).

**Figure 8:**
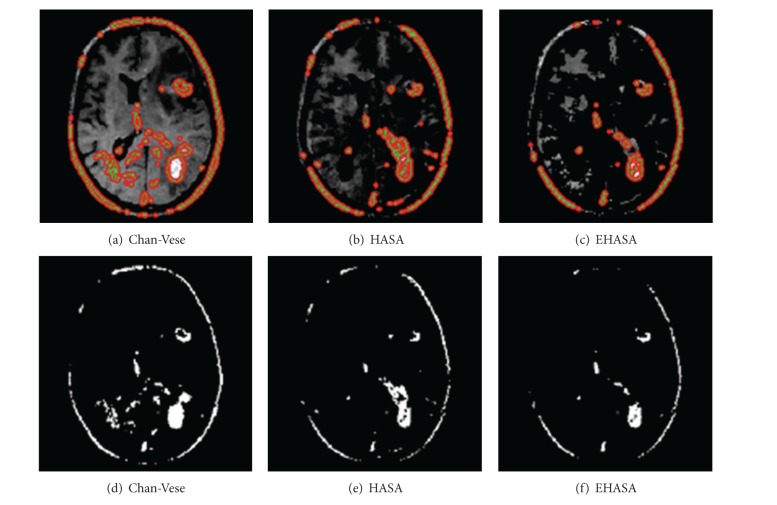
Result after the application of different techniques on input images shown in Figure 6(b).

For the sake of comparison we applied the method, given in [5], on the same data and the results we got are shown in Figure 5. It can be seen that both our methods perform better. The images shown in Figure 5 were resulted when we segmented by applying Chan-Vese method [1]. As can be seen, we got result two extra lobes and regions which are not tumors. In contrast, when we applied our methods the results were far better and improved. Similarly, for the Chan-Vese method, we put contour manually but in our method it is automatic and no user interaction is required. We took two other slices, shown in Figure 6, and subjected it to Chan-Vese, and our proposed techniques (HASA) and (EHASA). The results are shown in Figure 7 for Figure 6(a). and Figure 8 for Figure 6(b). It can easily be seen that our methods perform far better than that of Chan-Vese.

We selected two data sets; the MR images data set specification is shown in Table 3. These data sets were used to compare the processing time taken by Chan-Vese, HASA, and EHSA techniques. The measured results are listed in Table 4. From Table 4 it can be observed that Chan-Vese and HASA techniques take nearly close same processing time but EHASA takes more processing time as compared to Chan-Vese and HASA techniques. EHASA required finding first the binary image then mapping it to original image that required some processing time.

## 5. Conclusion

We proposed two techniques to overcome the problems with the existing techniques. Both techniques are based on symmetry. We have also compared our results with an existing technique. Our proposed techniques can identify the tumor/abnormality in either right or left side and can also find more than one tumor. These techniques do not require any user interaction and are fully automatic. One limitation of our techniques is that it will not give good results if the tumor is present on the symmetry line.

Although our proposed methods have addressed most of the identified problems but still it needs enhancement, we will do it in future so that we can get better segmentation results. We will implement these techniques in 3D.

## Figures and Tables

**Table 1:** Summary of pros and cons of supervised, unsupervised, and registration based segmentation.

	Supervised segmentation [2]	Unsupervised segmentation [3]	Registration based segmentation [4]
Pros	Can perform different task simply by changing the training set data. Learning is an essential component of this technique and when learning is completed then tumor will be detected automatically	No training required	Easy to make the comparison of two images

Cons	Special training required and, therefore, additional processing time required	Manual selection of region growing. Number of regions needs to be prespecified. Tumors have not clearly defined intensities	Registration may not be perfect due to different anatomical structure of the image and template

**Table 2:** Comparison of different segmentation techniques.

Techniques	Prior knowledge	User interaction required	Work in presence of noise	Simple
Supervised	Yes	Yes	Yes	No
Unsupervised	Yes	Yes	Yes	Yes
Registration	Yes	No	No	Yes
Symmetry	No	No	Yes	Yes

**Table 3:** Parameter values of different MR image data set.

Parameters	Data set 1	Data set 2
Magnetic field strength	1.5	1.5
File size	1049472	527858
Format	DICOM	DICOM
Width	1024	512
Height	1024	512
Bit depth	8	12
Color type	Grayscale	Grayscale
Modality	“MR”	“MR”
Samples per pixel	1	1
Photometric interpretation	Monochrome 2	Monochrome 2
Rows:	1024	512
Columns	1024	512
Pixel aspect ratio	(2 × 1 double)	(2 × 1 double)
Bits allocated	8	16
Bits stored	8	12
High bit	7	11
Pixel representation	0	0
Window center	127.5000	308.5015
Window width	255	536.3014

**Table 4:** Processing time comparison.

MR images data set specification	Chan-Vese [1] sec.	HASA sec.	EHASA sec.
Data set 1	43	46	52
Data set 2	45	47	55
